# Lithium Separation from Geothermal Brine to Develop Critical Energy Resources Using High-Pressure Nanofiltration Technology: Characterization and Optimization

**DOI:** 10.3390/membranes13010086

**Published:** 2023-01-09

**Authors:** Sutijan Sutijan, Stevanus Adi Darma, Christopher Mario Hananto, Vincent Sutresno Hadi Sujoto, Ferian Anggara, Siti Nurul Aisyiyah Jenie, Widi Astuti, Fika Rofiek Mufakhir, Shinta Virdian, Andhika Putera Utama, Himawan Tri Bayu Murti Petrus

**Affiliations:** 1 Chemical Engineering Department, Sustainable Mineral Processing Research Group, Faculty of Engineering, Universitas Gadjah Mada, Jl. Grafika No. 2, Kampus UGM, Yogyakarta 55281, Indonesia; 2 Unconventional Geo-Resources Research Center, Faculty of Engineering, Jl. Grafika No. 2, Kampus UGM, Yogyakarta 55281, Indonesia; 3 Geological Engineering Department, Faculty of Engineering, Universitas Gadjah Mada, Jl. Grafika No. 2, Kampus UGM, Yogyakarta 55281, Indonesia; 4 Research Centre for Chemistry, National Research and Innovation Agency (BRIN), Kawasan Puspiptek Building 452, Tangerang Selatan 15314, Indonesia; 5 Research Centre for Mineral Technology, National Research and Innovation Agency (BRIN), Jl. Ir. Sutami Km. 15, Tanjung Bintang 35361, Indonesia; 6 Balai Besar Logam dan Mesin, Ministry of Industry, Jalan Sangkuriang No. 12, Bandung 40135, Indonesia; 7 PT. Geo Dipa Energi, Jl. Dieng RT 01 RW 01, Desa Sikunang, Kabupaten Wonosobo 53456, Indonesia

**Keywords:** geothermal brine, green energy, high pressure, lithium, nanofiltration

## Abstract

There is a shift from internal combustion engines to electric vehicles (EVs), with the primary goal of reducing CO_2_ emissions from road transport. Battery technology is at the heart of this transition as it is vital to hybrid and fully electric vehicles’ performance, affordability, and reliability. However, it is not abundant in nature. Lithium has many uses, one of which is heat transfer applications; synthesized as an alloying agent for batteries, glass, and ceramics, it therefore has a high demand on the global market. Lithium can be attained by extraction from other natural resources in igneous rocks, in the waters of mineral springs, and geothermal brine. During the research, geothermal brine was used because, from the technological point of view, geothermal brine contains higher lithium content than other resources such as seawater. The nanofiltration separation process was operated using various solutions of pH 5, 7, and 10 at high pressures. The varying pressures are 11, 13, and 15 bar. The nanofiltration method was used as the separation process. High pressure of inert nitrogen gas was used to supply the driving force to separate lithium from other ions and elements in the sample. The research results supported the selected parameters where higher pressure and pH provided more significant lithium recovery but were limited by concentration polarization. The optimal operating conditions for lithium recovery in this research were obtained at a pH of 10 under a pressure of 15 bar, with the highest lithium recovery reaching more than 75%.

## 1. Introduction

The transportation and automotive industry is transitioning from internal combustion engine vehicles to electric vehicles (EV), with the primary goal of reducing CO_2_ emissions from road traffic. In this scenario, battery technology is at the heart of this transition, as it is an essential part of hybrid and all-electric vehicles’ performance, affordability, and reliability. In this scenario, lithium-ion batteries (Li-ion) play a crucial role due to their high gravimetric energy density (Wh/kg), volumetric energy density (Wh/L), and power density (W/kg) [1]. However, the growth of electronic mobility raises some new lithium availability problems, which are essential to finding alternative sources. Global electric car purchases increased to 6.6 million in 2021 from 3 million last year, meaning electric cars accounted for 9 percent of the market [2]. They were responsible for the increase in global car sales, which rose to 66.7 million last year, compared to 63.8 million in 2020. This phenomenon means that sales of non-electric cars fell by 700,000. An electric car lithium-ion battery contains approximately 8 kilograms (kg) of lithium. Global production of lithium reached 100,000 tons (90.7 million kg) last year, while international reserves were about 22 million tons (20 billion kg) [3]. However, all the lithium in the world cannot fit into the batteries of electric cars. The metal is also used in many other items, such as batteries for laptops and mobile phones, airplanes, trains, and bicycles [4].

Lithium resources are scattered in various places and can generally be found in seawater, minerals, clay, brine, and is also found in volcanic rocks and mineral springs [5]. Lithium is one of the minerals with different advantages in many areas, for example, in the industrial, energy, manufacturing, pharmaceutical, and economic sectors [6]. Along with the widespread use of lithium as part of renewable energy solutions, various effective methods have been introduced to extract lithium from its primary sources in nature.

Despite its wealth of natural resources, the abundance of lithium in nature is only 0.0018% [7], with Chile, the USA, China, and Australia, as the world’s largest lithium producer [8]. The global demand for lithium rapidly increases, and it is estimated that lithium consumption will reach more than 160,000 tons of lithium carbonate annually by 2025 [9]. Indonesia is not included in the list of the world’s top countries with lithium reserves. Still, it can extract lithium from its primary natural sources [10]. Lithium extraction can be carried out through various methods and from multiple sources: artificial sources of recycled electronic waste and lithium batteries [11] and natural sources from the environment consisting of naturally found minerals, salt lakes, underground water, or seawater [12]. Given a large number of salt stations in Indonesia, it is possible to extract lithium from bittern sources. Another source for lithium extraction is known to be geothermal brine. Studies show that geothermal brine contains many valuable minerals such as Si, Li, K, etc., that need to be removed. Dieng is one of the hot geothermal springs in Indonesia, which has been found to contain salt water with a lithium concentration of 4060 ppm [13]. This statement proves that geothermal salt water has the potential to be an alternative source of lithium in the future.

Some essential processes are ion exchange, solvent extraction, electrodeposition, and coprecipitation. Solvent extraction of lithium from bittern is a simple and effective method, but solvent recovery is energy intensive. Through the steam deposition method as the most straightforward method, the lithium contained in brine can be precipitated with a precipitating agent to form solid lithium aluminate [14]. However, considering that the magnesium ion in bittern is challenging to separate from the lithium-ion, the efficiency of this method for producing lithium metal is not yet known [15].

Other methods include membrane technology, such as membrane distillation (MD) technology [16] and electrodialysis [17]. The membrane distillation method involves a thermal membrane separation process whereby volatile compounds are transferred from a hot aqueous solution (usually saline water) through a microporous hydrophobic membrane due to the driving force between partial pressure caused by the temperature difference from both sides of the membrane [18]. However, fouling is a common problem in membrane distillation as it inhibits desalination membrane activity. Fouling can occur by three main factors and interactions between particles: the membrane material’s nature, the solute’s nature, and the operational parameters [19]. Contamination of the membrane can reduce the water production rate and contamination can also damage the membrane because it requires frequent cleaning [20]. Electrodialysis is also an alternative to lithium extraction structured within an electrodialysis stack employed with direct current, which involves the ion transfer through a membrane with electric potential charge difference as the driving force [17]. The distillation membrane mostly serves to concentrate the metal content in the solution [16], while electrodialysis still has difficulties in separating magnesium and lithium ions with almost the same hydration radii [21]. So, nanofiltration is an alternative technology to obtain high concentrations of lithium because, theoretically, only monovalent ions can pass through the membrane [21].

Nanofiltration is another method of extracting geothermal brine, a source of lithium in the form of water that comes from deep in the earth and contains many minerals because it is in contact with geothermal heat and flows between rocks in the world. This method is possible considering that Indonesia is located on the Pacific Ring of Fire, with one hundred and twenty-nine active volcanoes, providing abundant geothermal energy. Indonesia’s geothermal potential is estimated at 40% of the global potential, or 29 GW [22]. Geothermal brine in the geothermal environment contains highly dissolved salts, corrosive chlorine, and sulfate ions. The number of these ions is relative compared to carbonate and bicarbonate. The chemical composition of brine is sodium (Na), potassium (K), magnesium (Mg), calcium (Ca), chlorine (Cl), sulfate (SO_4_), silicate (SiO_2_), and carbonic acid (HCO_3_). This research will make synthetic geothermal brine by mixing demineralized water with a heated salt solution containing a concentration of lithium (350 mg/L), sodium (63,570 mg/L), potassium (21,370 mg/L), magnesium (12 mg/L), and calcium (43 mg/L) with the addition of an acid or base solution to be tested by the nanofiltration method.

In this study, nanofiltration as a separation method is carried out in the liquid phase with a driving force as an alternative to reverse osmosis and ultrafiltration processes. The nanofiltration method with high pressure provides a higher driving force so that the molecules in the solution will be more easily separated from the geothermal brine solution through the membrane pores. Nanofiltration uses cross-flow membranes with high flow rates to increase permeability and reduce potential contamination. The injected dissolved particles (such as dissolved salts) are separated from the wastewater and do not accumulate on the membrane surface [23]. Nanofiltration membranes with pore sizes as small as 0.001 μm are limited in processing raw water into drinking water. Nanofiltration membranes are more helpful in separating water from dissolved solids, bacteria, viruses, multivalent ions such as Ca^2+^, Mg^2+^ which cause hardness, or molecules with a molecular weight of about 200–5000 but cannot separate monovalent ions such as Na^+^, K^+^ and others. The nanofiltration membrane can only treat raw water, such as freshwater [24]. However, nanofiltration offers significantly better retentions than ultrafiltration for separating small molecules such as sugars, amino acids, peptides, and even ions with higher fluxes than reverse osmosis [25].

Furthermore, lithium ions (Li^+^) can penetrate the monovalent ion exchange membrane from the brine (feed) into the concentrate (purified solution) under a high-pressure driving force. However, the ionic radius of lithium is similar to other ions, such as magnesium Mg^2+^ and calcium Ca^2+^ [26], thereby limiting the development of technologies for lithium extraction from geothermal brine. To selectively separate the lithium ions from the other ions, the ion exchange membrane must be highly permeable to counter ions but impermeable to co-ions [27]. Smaller ions are thought to permeate selectively through membranes with highly cross-linked structures compared to larger ions [28]. Additionally, the effects of pH on membrane permeability were quite complicated and therefore were observed to identify the impact of pH upon the presence of hydroxide ions within the solution, which could affect the osmotic pressure, effective driving force, electrostatic–ion interaction, and membrane permeability [25]. Therefore, appropriate methods for recovering lithium from other ions through varying experimental conditions were urgently required.

Nanofiltration membrane technology NF2, similar to the NF245, provides a suitable separating method for recovering lithium from geothermal brines, providing appropriate pore sizes, stable ion rejection of at least 90% with an average flux rate of 42 LMH and the ability to selectively separate monovalent and divalent/multivalent ions [26]. Nanofiltration membrane is also known to be energy efficient as well as environmentally-friendly [25]. This study investigated lithium’s recovery from geothermal brine under various acidic and basic conditions under high pressure using an NF90 nanofiltration membrane. The experiments were carried out under pH values of 4.7 and 10 under high pressure of 11, 13, and 15 bar.

## 2. Materials and Methods

### 2.1. Nanofiltration Membrane

This research and observation employed a nanofiltration membrane NF2 with a 99% stable rejection average flux rate of 55 LMH from Rising Sun Membrane Technology (Beijing) Co., Ltd., Shunyi District, Beijing, China. The nanofiltration membrane is produced from polyamide material. The membrane was carried out in an experiment within a room temperature range of 25 ${}^{\circ }$C, with an operating temperature range of 2–45 ${}^{\circ }$C, an operating pH range of 2.0–11.0, a maximum working pressure of 41 bar, and a minimum salt rejection of 99%.

### 2.2. Feed and Recovery Solution

#### 2.2.1. Feed

This experiment uses a synthetic geothermal brine composed of several dissolved chloride salts in demineralized water to create a similar brine composition to that of the natural geothermal brine from Dieng, Central Java, Indonesia. The composition used follows the composition in Table 1.

The synthesized geothermal brine with the composition from Table 1 does not include silicon, and several other components are usually present in a natural geothermal brine from Dieng. Therefore, it was assumed that the feed had undergone pretreatment for the removal of impurities by methods of emulsion, suspension, and precipitation. Pretreatment is important because silica is the biggest problem in extracting precious metals using membranes. Several studies have been carried out on removing silica content in geothermal brine to avoid fouling phenomena [29,30].

#### 2.2.2. Nanofiltration System

**Figure 1:**
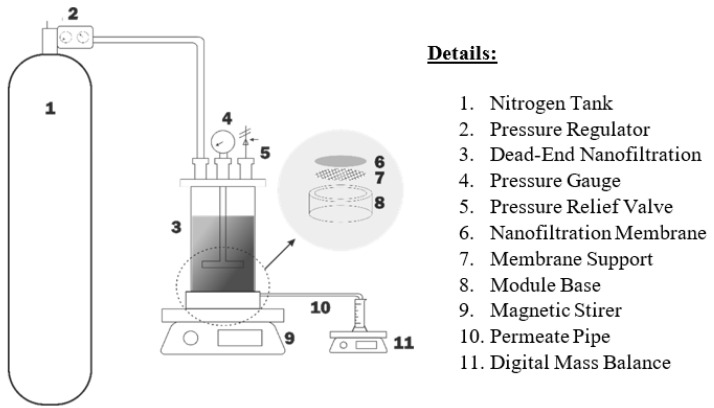
Experimental apparatus for nanofiltration.

The nanofiltration system was carried out on a laboratory scale using a Dead-End Module (HP4750 Stirred Cell) made from stainless steel from Sterlitech Corporation. The HP4750 Stirred Cell has an active membrane area of 14.6 cm^2^ (2.26 in^2^) with a maximum processing volume of 300 mL but operates with only 100 mL processing volume. The experimental apparatus for nanofiltration is shown in Figure 1. The experiment was carried out at room temperature of 25 ${}^{\circ }$C, with an operating pressure of 11, 13, and 15 bar, varied amongst pH values of 4, 7, and 10. The weight of permeate collected and flux rate was recorded every 5 min until a permeate volume of 75 mL was reached. The samples before and after the nanofiltration process were analyzed using ICP-OES.

### 2.3. Theoretical Model

During the separation process by the nanofiltration system, the transfer of ions and solution is calculated respectively with time, also known as ionic flux and solution flux. Solution flux, transmembrane flux, commonly known as total volume flux, denoted by $J_{v}$ $\left(\frac{L}{m^{2}\cdot h}\right)$, is the transfer of specific solution through a membrane per unit of time and area as formulated by the following equation [31]:

(1) $$J_{v}=\frac{V_{p}}{A\cdot t}$$

Index $V_{p}$ is the permeate volume *L*, *A* is the effective membrane surface area $m_{2}$, and *t* is time *h*. Ionic flux or molar solute flux is denoted by $J_{s}$ $\left(\frac{gr}{m^{2}\cdot h}\right)$ which is the rate of transfer of specific ions through the membrane per unit of time and area, which can be formulated by Equation (2) whereby the initial concentration is denoted $C_{0}$ $\left(\frac{gr}{L}\right)$, and the concentration at a specific time, *t*, is indicated as $C_{t}$ $\left(\frac{gr}{L}\right)$ [32]:

(2) $$J_{s}=\frac{V_{p}\cdot \left(C_{t}-C_{0}\right)}{A\cdot t}$$

The mass balance and composition balance proposed for the nanofiltration model represents the flow rate of the feed, permeate, and retentate as indicated by the following equation [33]:

(3) $$Q_{F}=Q_{R}+Q_{P}$$

(4) $$C_{Fi}\cdot Q_{F}=C_{Ri}\cdot Q_{R}+C_{Pi}\cdot Q_{P}$$

(5) $$Q_{P}=J_{V}\cdot A$$

(6) $$J_{V}=L_{P}\cdot \left(\Delta P-\Delta \pi \right)$$

$Q_{F}$ is the feed flow rate, $Q_{R}$ is the retentate flow rate, and $Q_{P}$ is the permeate flow rate. Index $C_{F}$ is feed concentration, $C_{R}$ is the retentate concentration, $C_{P}$ is the permeate concentration, Δ*P* is the pressure difference applied across the membrane, Δπ is the osmotic pressure difference across the membrane, and $L_{P}$ is the pure water permeability.

From the mass balance Equation (3), the percentage recovery of the specific ion R_i, which represents the number of ions recovered from the feed after going through the nanofiltration process, can be calculated using the following equation [26,33,34]:

(7) $$Q_{F}=Q_{R}+R_{Li}\cdot Q_{F}$$

(8) $$Q_{R}=Q_{F}-R_{Li}\cdot Q_{F}$$

(9) $$Q_{R}=1-R_{Li}\cdot Q_{F}$$

(10) $$R_{Li}=1-\frac{Q_{R}}{Q_{F}}$$

(11) $$R_{Li}=\frac{Q_{P}}{Q_{F}}$$

(12) $$R_{Li}\left(\%\right)=\left(1-\frac{Q_{R}}{Q_{F}}\right)\times 100\%$$

where $R_{Li}$ is the recovery of lithium-ion. However, an alternative method to calculate the percentage of recovery of lithium ions may use the following Equation (13), where $V_{t}$ is the volume at *t*, $V_{0}$ is the initial volume, $V_{F}$ is feed volume, and $C_{F}$ is feed concentration [34]:

(13) $$R_{Li}\left(\%\right)=\left|\frac{\left(V_{t}\cdot C_{t}-{V_{0}\cdot C}_{0}\right)}{V_{F}\cdot C_{F}}\right|\times 100\%,$$

## 3. Results and Discussion

This study was carried out using lithium extraction by nanofiltration process under high pressure of 11, 13, and 15 bar with varying pH solutions of 4, 7, and 9 to determine the success of the process. The variations between two independent variables of pH and pressure were needed to study the effects upon the process’s solution flux, ionic flux, and efficiency recovery. The results were then optimized to determine the ideal operating conditions needed to recover lithium from artificial geothermal brine.

### 3.1. The Effects of Operating Condition Pressure and pH on the Solution Flux, J_v_

**Figure 2:**
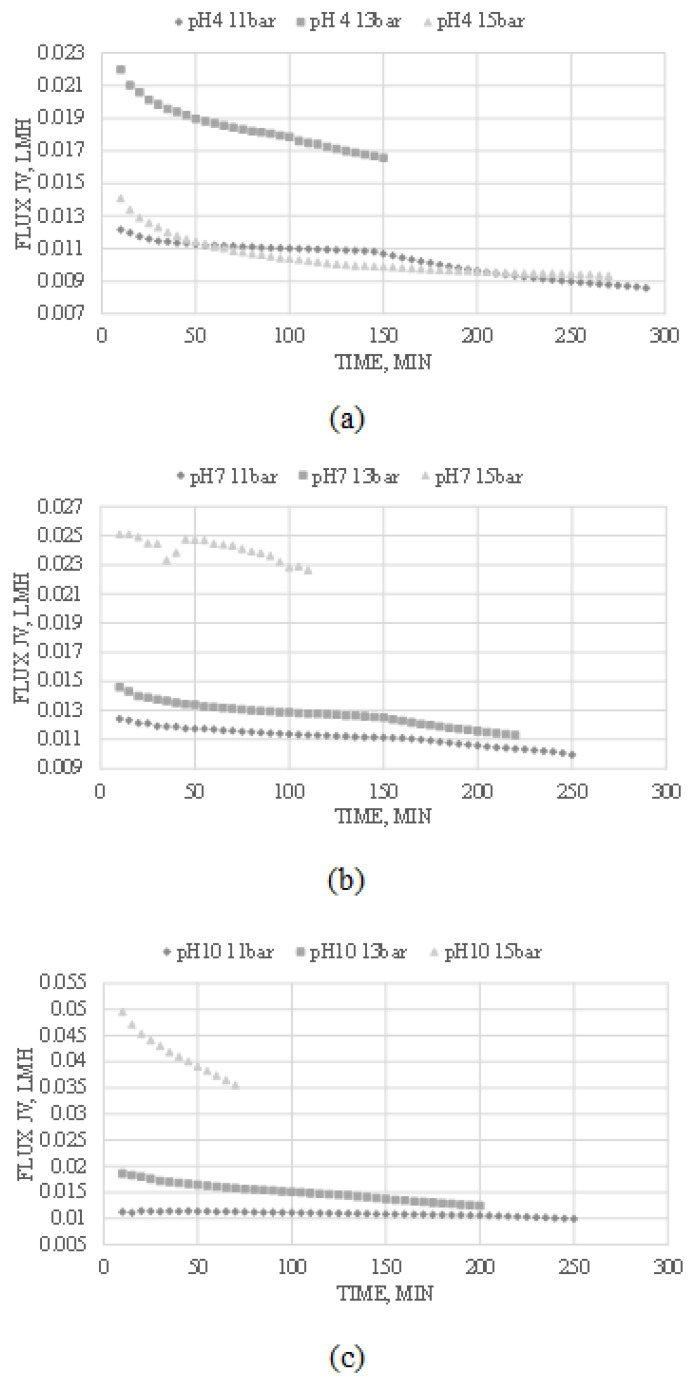
Effect of operating conditions on solution flux by nanofiltration separation at (**a**) pH 4; (**b**) pH 7; (**c**) pH 10.

Solution flux, also known as the permeate flux, indicates the flow and volumetric flow rate of the permeate that passes through the nanofiltration membrane per unit area per unit of time. The varying operating conditions have differential effects on the permeate flux, indicating the membrane’s performance in ion extraction and rejection. The impact of the varying operating conditions on the permeate flux in this study is shown in Figure 2.

As can be seen from Figure 2, the permeate flux increases with an increase in pressure applied to the system. An increase in pH upon the sample also increases the permeate generally, decreasing the retention time within the system. Furthermore, the flux was highest at the start of each experiment. It later reduced over time due to membrane blockage by the ions in the system, eventually resulting in membrane fouling [35]. The membrane fouling was caused by the accumulation of larger ions within the membrane pores. The continuous expansion will cause the layers on the surface membrane to become denser and more compact, decreasing the membrane’s porosity and the solution and lithium-ion flux [36]. Figure 2c, with operating conditions of pH 10, shows the highest flux rate, and Figure 2b follows after, with Figure 2a coming in last. Figure 2 shows a trend that an increase in the pH of the solution will result in higher flux. This phenomenon happens because the nanofiltration membranes are usually charged membranes, and the charge density depends on the pH of the contacting solution [37].

The nanofiltration membrane, in general, is negatively charged at all pH values. However, the fixed charge density of the membrane decreases gradually when the membrane is in contact with a feed solution with a lower pH value. At lower pH values, the charge density decreases, which causes partial dissociation of the carboxylic acid groups within the membrane matrix and decreases the hydrophilicity, resulting in higher membrane resistance and lower flux [37]. Therefore, a basic feed solution at a higher pH value shows higher flux than an acidic feed solution at lower pH because the effects of pH have definite effects on the fouling potential of the membrane.

Additionally, Figure 2b,c show that an increase in pressure leads to an increase in flux. This event happens due to the increased driving force applied to the system. The increase in pressure upon the system provides an increased driving force, which results in increased permeate flux. However, Figure 2a does not follow the same trend as Figure 2b,c. The flux of Figure 2a shows a permeate flux within a feed solution with an acidic pH value of 4. Within an acidic environment, the membrane resistance increases, which causes the membrane pores to clog. This phenomenon eventually leads to the accumulation of specific ions near the boundary layer of the membrane surface, causing concentration polarization and incrustation [38]. The concentrated ion layer causes the osmotic pressure along the surface of the membrane to increase, causing a decrease in net driving force and resulting in lower flux [39]. Thus, by analyzing the results shown by the different trends of the solution flux, it can be concluded that the best operating condition to extract lithium by nanofiltration is at an operating temperature of 15 bar at a feed solution pH of 10, which gave the highest solution flux compared to other operating condition results.

### 3.2. The Effect of Operating Condition Pressure and pH on the Ionic Flux, J_s_

**Figure 3:**
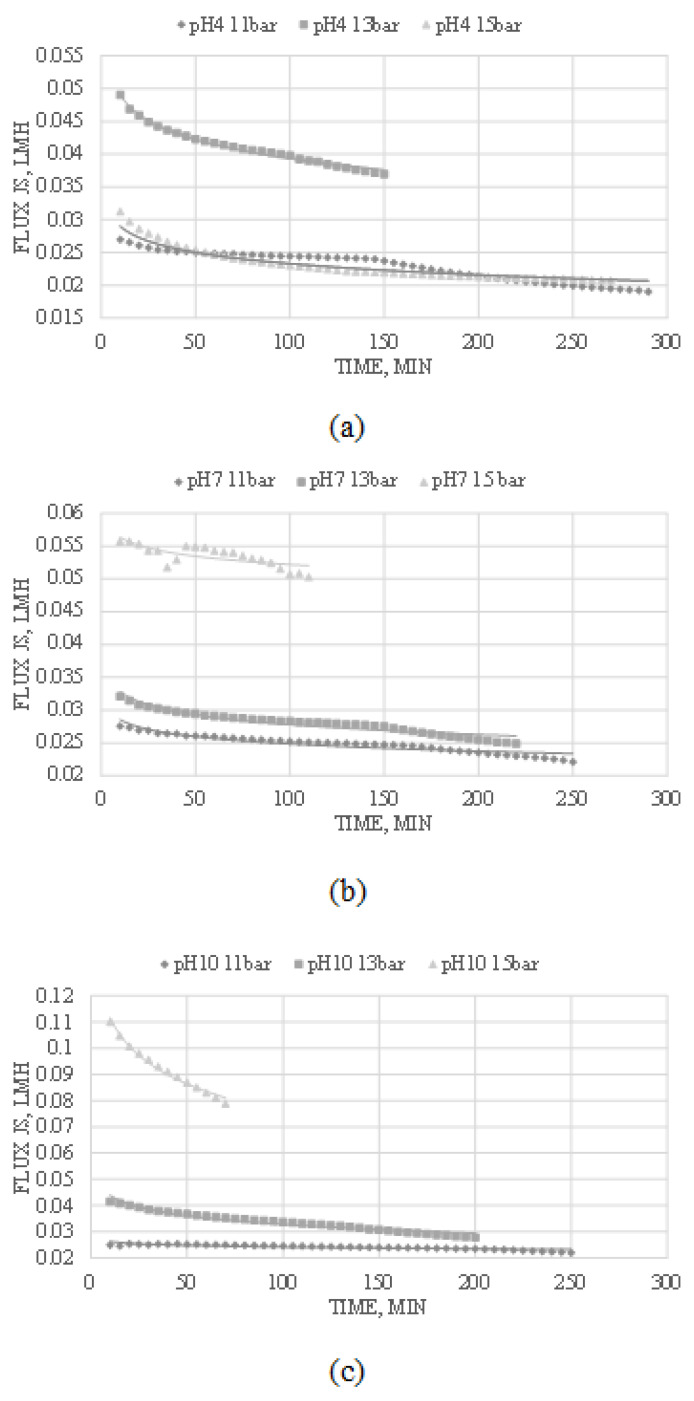
Effect of operating conditions on ionic flux by nanofiltration separation at (**a**) pH 4; (**b**) pH 7; (**c**) pH 10.

Ionic flux indicates the ions’ flow and volumetric flow rate that passes through the nanofiltration membrane per unit area per unit of time. The varying operating conditions gave different results and are shown in Figure 3. Based on Equation (2), a specific trend was drawn on the various figures in Figure 3 to compare the ideal ionic flux to the experimental ionic flux.

Figure 3a–c show that the ionic flux increases with increasing operating pressure. This phenomenon happens due to the increase in driving force by increasing pressure. An increase in pressure results in a greater convective rate of solutes towards the membrane surface [38]. However, all figures in Figure 3 show that the ionic flux decreases over time due to a potential membrane blockage. This membrane blockage happens when several ions with large ionic radii are present in the solution and cannot pass through the porous membrane, which causes the membrane to clog. The order of the ionic radii or also known as cationic hydration radii, in the solution follows [26]: Mg^2+^ > Ca^2+^ > Li^+^ > Na^+^ > K^+^ as shown in Table 2. Sodium ions and potassium ions have smaller hydration radii than lithium ions and therefore have higher mobility. They can diffuse faster than lithium ions across the membrane even though these ions have lower charges than their co-ion counterparts [25].

Another major ionic flux factor would be the feed solution’s pH value. The ionic flux is much higher for feed solution with a higher pH value than for a lower pH value. At a solution with a higher pH value, the cations Mg^2+^, Li^+^, and K^+^ are more dominant below IEP (isoelectric point, pH value where molecules have zero net electric charges) while the anions Cl^−^ determine the salt rejection. Additionally, in a solution with a lower pH value, the anions are more dominant above IEP, while the cations and membrane decide the salt rejection [25]. This phenomenon happens because the membrane is less negatively charged at a lower pH value. Therefore, the electrostatic repulsion between the membrane and cations determines the ion rejection, whereas the membrane is more negatively charged at higher pH. Ion rejection is determined by the repulsion between anions and the membrane [40].

Moreover, it can be seen from Figure 3b that at a pH solution of 7 with a net charge close to IEP, the ionic flux decreases steeply at the operating condition of 15 bar but rises again over time, thus creating a V shape curve. This event may occur due to the fouling of the membrane. Ionic flux is suspected to be lowest at the IEP of the feed and increases when the pH is adjusted away from the dominant charge of the ions [38]. At an operating pressure of 15 bar, the flux increases after a steep decrease due to the constant contact between the ions and the membrane under a high pressure which causes a high driving force. Moreover, an increase in driving force increases the permeate flow by producing higher turbulence which causes dispersion within the solute molecules near the membrane surface, reducing the layer thickness of the membrane and countering the concentration polarization. The high shear rates generated from the high driving force near the membrane surface cause the accumulated layers of ions to strip, reducing the hydraulic resistance of the membrane fouling [38,41].

From Figure 3c, the ionic flux is the highest compared to the other operating conditions. This experiment was carried out under a pH of 10, indicating that the membrane was more negatively charged than the usual system. Additionally, the cations in the solution were dominant at a higher pH value. The flux was higher due to the smaller ionic radii providing higher mobility than their counter-ions. This effect leads to a lower rejection of salts containing monovalent ions, whereas salts containing multivalent ions were efficiently rejected by the membrane [40]. Hence, the higher flux indicated lower rejection and mobility of the monovalent cations, as seen in Figure 3c.

**Figure 4:**
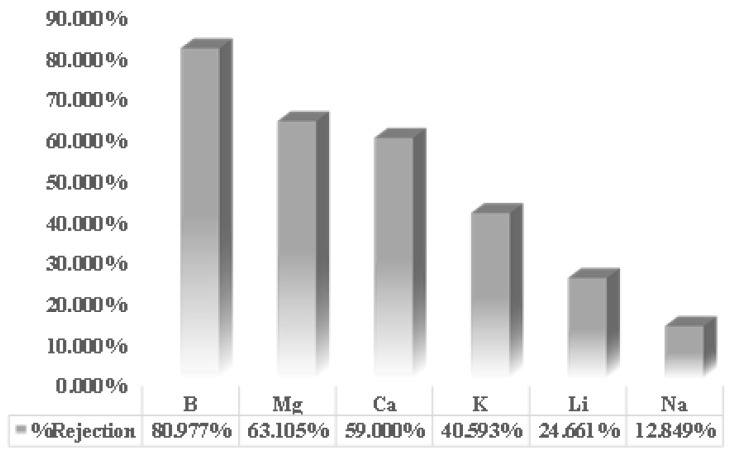
The percentage of rejection of several ions from research results with variations in the operating voltage.

**Figure 5:**
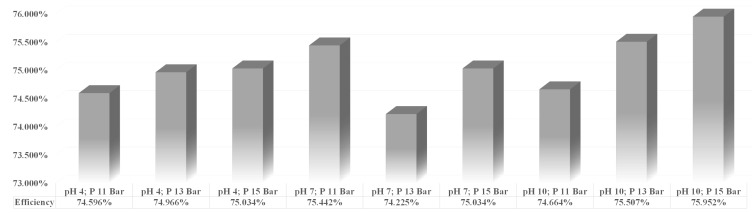
Efficiency of lithium recovery at various operating conditions.

Figure 4 shows the rejection values for each ion contained in geothermal brine. Theoretically, the larger the radius of an ion, the more difficult it is for the ion to pass through the membrane. However, the results showed that boron, which has the smallest ionic radius, actually had the most significant rejection percentage compared to other ions. This phenomenon is because the measured ions are in the liquid, so the radius that must be used as the primary reference is the hydrated radius. Therefore, when viewed from the size of the hydrated radii in Table 1, the order of the radii of each ion is B > Mg > Ca > K > Li > Na. This phenomenon is in line with the research results depicted in Figure 5.

### 3.3. Optimizing Parameters by Application

The previous explanation regarding the effects of pH and pressure on the solution and ionic flux was further analyzed using Equation (10) and lithium concentration data from ICP-OES in calculating the percentage recovery of lithium in various operating conditions.

The results seen from Figure 3 and Figure 4 regarding solution flux and ionic flux correspond to the lithium recovery. The increase in the flux of lithium ions dramatically influences the increase in the percentage of lithium recovery, which indicates that various operating conditions have multiple effects on the recovery of lithium by the nanofiltration process [4]. Figure 5 shows the percentage of lithium recovery under different operating conditions conducted in this study. Based on Figure 5, the results clearly showed that an increase in pH increases lithium recovery. Likewise, the increase in pressure increases lithium recovery.

However, there was an anomaly regarding the general trend for the samples analyzed within the solution of pH 7. The lithium recovery for pH 7 did not increase as pressure increased, which may have been caused by different fouling formations due to the other ions present within the samples. This phenomenon occurs for the pH 7 solution because the membrane’s charge was assumed to be close to the isoelectric point of the ions. Hence the membrane was less negatively charged, and the flux depended on the number of monovalent and divalent ions present within each solution [25]. It was suspected that sample 5 (pH 7; 13 bar) had more divalent cations (Mg^2+^, Ca^2+^), which were capable of forming bonds with the carboxylic groups present on the surface of the membrane, providing linkage for fouls towards the membrane surface, hence accelerating fouling formation [25]. Continuous fouling build-up within the membrane surface will cause the layers on the surface membrane to become denser and more compact, which will decrease the porosity of the membrane, hence reducing lithium-ion flux and lithium recovery [36].

Generally, most NF membranes are negatively charged when operating in an aqueous environment. The nanofiltration membrane exhibited a negative zeta potential at neutral pH. At lower pH values, the zeta potential is higher (less negative) [42]. The sulfonic acid groups (-SO_3_-) present in most nanofiltration membranes are strongly acidic and completely dissociate over almost the entire pH range, whereas the carboxyl groups (-COO-) exist. Like cellulose acetate membranes, it is a weak acid and does not dissociate at low pH [43]. Polyamide membranes may also contain positively charged ammonium groups (NH3 is positively charged only in acidic media, whereas -R3N is positively charged over the entire pH range) [42,43].

**Figure 6:**
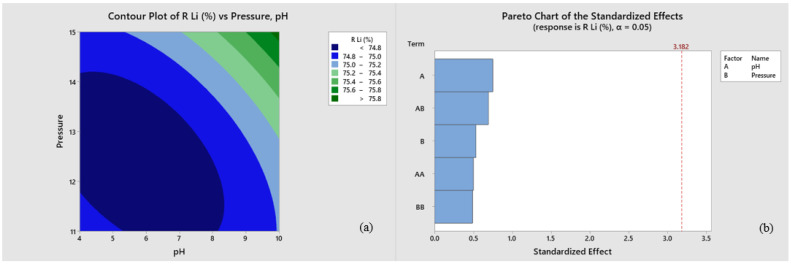
Statistical approximation diagram of standard effects for optimizing experimental variables. (**a**) Contour diagram. (**b**) Pareto diagram.

Figure 6 compares the experimental factor variables to the lithium recovery percentage. The percentage value of lithium recovery was calculated using Equation (10) and optimized using the Minitab Statistical Software application to obtain the Pareto graph in Figure 6. Furthermore, Figure 6a shows a contour plot representing the relationship between factor variables (pressure and pH) and lithium percentage recovery from geothermal brine using the nanofiltration method. An increase in pH results in a greener region, indicating a more significant recovery percentage. Similarly, an increase in pressure shows a light blue color, almost close to the green, indicating an increase in the recovery percentage. From the description, the weaker the blue or closer to the dark green, the better the lithium recovery from each brine solution.

Figure 6b shows a Pareto chart. The Pareto chart shows which factors have a significant effect in Figure 6b shows that the phenomena described in Figure 6a that pH and pressure significantly impact the study. Figure 6b also indicates that although pH and pressure play a dominant role in lithium recovery, pH has a more significant role than pressure. This phenomenon is reinforced by the phenomenon depicted in Figure 3a and Figure 4a, that an increase in pressure does not always give higher ionic and solution flux.

In theory, the greater the pressure exerted on the membrane, the greater the driving force, hence the lower the retention rate of lithium ions, leading to more remarkable recovery [26]. For increasing pH solution, the membrane becomes more negatively charged, causing higher rejection and repulsion of the anions due to counter charged surface, leading to lower salt retention containing monovalent anions and cations and higher rejection of multivalent anions [40,44].

The solution and ionic flux were lower than the other results at a lower pH feed solution and pressure. The lithium recovery may be around an expected value of 74% to 75%, however, required more time to achieve the inevitable lithium recovery. With lower feed flow, the possible build-up of membrane fouling was more significant as concentration polarization was more likely to happen at a low feed flow rate [4]. Increased pressure was required to overcome the fouling layer build-up on the membrane surface. The increase in pressure provides a higher driving force and feed flow rate, which causes the deformation of the layers built on the surface of the membrane. As a result, more ions, including monovalent and divalent ions, are continuously forced through the membrane leading to less ion retention. Still, the larger ions, Mg^2+^ and Ca^2+^, are abundant in the permeate collected [45]. In short, concentration polarization will more likely occur in lower pH value and lower pressure, and salt leakage will potentially happen in this phenomenon when increased pressure is applied [4,46].

So, based on the statistical approach and its conformity with the phenomena mentioned above, it can be concluded that the best-operating conditions of this study for lithium recovery were achieved at pH 10 under 15 bar pressure, with lithium recovery reaching more than 75%. This result is in line with the research by Liu et al. [42], which states that most nanofiltration membranes have a lower zeta potential value at a higher pH. The lowest zeta potential value at pH 10 is in most nanofiltration membranes, so the membrane is the most negative under these conditions. As a result, the more positively charged lithium ions pass through the membrane, the higher the recovery obtained in this operating condition [42].

## 4. Conclusions

This study shows that pH and pressure are essential parameters in lithium recovery from geothermal brine, artificial geothermal brine, specifically, in this research, without any silicon and other components, as it was assumed that the feed had undergone pretreatment for the removal of impurities. The increased pH provided a more negatively charged membrane, hence more significant rejection against anions and lower salt retention containing monovalent and divalent cations. In contrast, increasing pressure provided a greater driving force for higher feed flow, leading to greater solution flux, ionic flux, and lithium recovery. The solution and ionic flux decrease until a stagnant rate is reached. The decrease was mainly caused by concentration polarization and membrane fouling. The increase in pressure would provide greater driving force and contact with the layer on the membrane surface to overcome membrane fouling but may lead to salt leakage. The research results supported the selected parameters where higher pressure and pH provided more significant lithium recovery but were limited by concentration polarization. The optimal operating conditions for lithium recovery in this research were obtained at a pH of 10 under a pressure of 15 bar, with the highest lithium recovery reaching more than 75%.

## Figures and Tables

**Table 1:** Synthetic brine geothermal composition.

Component	Concentration [ppm]	or	Concentration [M]
Na^+^	7120		0.310
K^+^	2200		0.060
Mg^2+^	107		0.004
Ca^2+^	401		0.010
Li^+^	39		0.006
B^3+^	305		0.031

**Table 2:** Hydration radii of various ions in the salt solution [26].

Ion	Li^+^	Na^+^	K^+^	Mg^2+^	Ca^2+^	B^3+^
Rh (nm)	0.382	0.358	0.331	0.428	0.412	0.555

## Data Availability

Not applicable.
